# Genomic epidemiology of *Chikungunya* virus in Colombia reveals genetic variability of strains and multiple geographic introductions in outbreak, 2014

**DOI:** 10.1038/s41598-019-45981-8

**Published:** 2019-07-10

**Authors:** Yeneiris Villero-Wolf, Salim Mattar, Andrés Puerta-González, German Arrieta, Carlos Muskus, Richard Hoyos, Hernando Pinzon, Dioselina Peláez-Carvajal

**Affiliations:** 1 Instituto de Investigaciones Biológicas del Trópico, Universidad de Córdoba, Montería, Córdoba Colombia; 2 Clínica Salud Social, Sincelejo, Sucre, Colombia; 3 Universidad de Antioquia, Medellín, Antioquia Colombia; 4 Grupo de Salud Pública, Corporación Universitaria del Caribe-CECAR, Sincelejo, Sucre, Colombia; 5 Programa de Estudio y Control de Enfermedades Tropicales (PECET), Facultad de Medicina, Universidad de Antioquia, Medellín, Antioquia Colombia; 6 Grupo de Investigación en Resistencia Bacteriana y Enfermedades Tropicales, Universidad del Sinú, Montería, Córdoba Colombia; 7 Universidad de Cartagena, Hospital Infantil Napoleon Franco, Cartagena, Colombia; 8 Grupo de virología, Instituto Nacional de Salud, Bogotá, DC Colombia

**Keywords:** Phylogenetics, Alphaviruses, Molecular biology

## Abstract

Chikungunya virus (CHIKV) is considered a public health problem due to its rapid spread and high morbidity. This study aimed to determine the genetic diversity and phylogenetic relationships of CHIKVs in Colombia. A descriptive and retrospective study was carried out using sera of patients infected with Chikungunya during the outbreak in Colombia. The whole genomes of CHIKV (n = 16) were sequenced with an Illumina Hi-seq 2500 and were assembled using the Iterative Virus Assembler software. A Bayesian inference phylogenetic analysis was carried out with 157 strains of worldwide origin. The Colombian CHIKV sequences were grouped in the Asian genotype; however, three independent phylogenetic subclades were observed, probably the result of three separate introductions from Panama, Nicaragua, and St. Barts. Each subclade showed several different non-synonymous mutations (nsP2-A153V; nsp2-Y543H; nsp2-G720A; nsP3-L458P; Capside R78Q), that may have functional consequences for CHIKV biology and pathogenesis. These same mutations may affect the efficacy of potential CHIKV vaccines.

## Introduction

Chikungunya virus (CHIKV) is an alphavirus of the Togaviridae family, that is transmitted by the bite of mosquitoes of the species *Aedes aegypti* and *A*. *albopictus*^1^. CHIKV is considered a public health problem due to its rapid spread and high morbidity^2^. It causes a febrile illness accompanied by maculopapular rash and severe joint pain that can last for months or years^1^. The incapacitating nature of the disease has caused a substantial economic burden and collapse in health systems^1^. The local transmission of CHIKV has been reported in more than 100 countries and territories in Asia, Africa, Europe, and the Americas^1^. According to the World Health Organization (WHO), between December 2013, and 2017, the islands of the Caribbean and the Americas registered >2,5 million suspected and confirmed^3^.

The study of whole genome sequences of CHIKV isolates from different regions of the world has facilitated an understanding of the evolutionary history of this virus^4^. The first phylogenetic studies of CHIKV identified three geographically associated genotypes: West Africa (WA), East/Central/South Africa (ECSA) and Asia (AS)^5^. However, in the years 2005–2006 a new descendant from the ECSA, the Indian Ocean lineage (IOL) was described^6^. IOL developed a higher affinity for the *Aedes albopictus* vector, resulting from the E1-A226V, E2-I211T, E1-T98A and E2-L210Q mutations^2^, and was associated with the massive epidemics of 2005–2006 in the Indian Ocean Islands and the Indian subcontinent^6,7^. Since the outbreaks in India and the Indian Ocean, CHIKV has spread worldwide, and the locations of epidemics have changed drastically^8,9^. In 2007, the first outbreak of transmission in Europe was reported in Italy and in 2013, the first cases in the Caribbean island of Saint Martin^10^. In January 2015 CHIKV was disseminated to 42 countries or territories in the Caribbean, Latin America and North America (Florida)^11,12^.

In recent decades, Colombia has suffered epidemics due to a large number of emerging arboviruses such as dengue virus, Venezuelan equine encephalitis virus, CHIKV, and Zika, which have caused severe public health problems^13^. The first case of local transmission of CHIKV in Colombia was notified in August 2014 in the department of Bolivar^14^. According to the Pan American Health Organization, Colombia was the country with the third highest number of cases after Brazil and the Dominican Republic^15^; between 2014 and 2015 there were 460,484 cases diagnosed by clinical symptoms and 4,658 confirmed by the laboratory^16,17^. Finally, 21,149 cases have been notified between 2016 and August 2018, demonstrating the endemicity of Chikungunya in Colombia^18^.

The rapid distribution of CHIKV could be related to the mobility of people and merchandise. According to the National Institute of Health (INS), in Colombia, the first reported cases were imported from Venezuela, the Dominican Republic and Panama^19^. Previous phylogenetic analyses with partial sequences of genes nsP1, E1, and E2 in Colombia, found that CHIKV was similar to the Asian genotype and was related to isolates from Saint Martin Island, Virgin Islands, Mexico and Brazil^14,20^. However, these phylogenetic studies did not analyze the complete genome or sequences of many countries affected by CHIKV, which precluded a comprehensive evaluation of the genetic variability, and phylogenetic relationships of the strains studied^21^.

Previous studies have determined that nucleotide substitution rates occur more frequently during urban transmission than in enzootic transmission^21^. It is known that the Asian genotype circulates mainly in an urban transmission cycle and has a genomic evolution rate of 4.71 (95% BCI: 3.84–5.65) nucleotide substitutions per year, higher than the genotype. ECSA (2.31, 95% BCI: 1.89 to 2.71)^21,22^. There is evidence that some Asian strains in the Colombian Caribbean are already exhibiting nucleotide substitutions in proteins nsP1 and E2 20, but it is unknown if there are variations in other regions of the viral genome that are associated with autochthonous vectors or the clinical manifestations of patients.

The present study aimed to determine the genetic diversity, phylogenetic relationships, and possible routes of introduction of CHIKV strains isolated in Colombia and the Americas.

## Results

### Viral isolation

CHIKV was isolated from 96.5% (54/57) of the sera, 65.4% (n = 36/54) of the isolated viruses coming from Cartagena, 29% (n = 16/54) from Ovejas, 1.8% from Planeta Rica and 1.8% from San Joaquín-Mahates (SJ-Mahates). Positive cultures were obtained from samples with viral loads of 1.19 × 10^2^ and 1, 25 × 10^7^ viruses/mL, which generally correspond to the first four days of the acute phase of the disease. In the three sera from which viral isolation was not achieved, the viral loads were less than 4.5 × 10^1^ and corresponded to the fifth day of disease. The three strains donated by the Colombian National Institute of Health (INS) were isolated in C6/36 and Vero cells. The most marked difference between the Vero cell line and C6/36 was the cell lysis and the time of appearance of the cytopathic effect (CPE). It was observed that the CPE in C6/36 cells occurred from the fifth day with the formation of syncytia. The C6/36 cell monolayer remained stable for approximately seven days. In contrast, in the Vero cells, the CPE was observed at 24–48 hours post-infection (hpi) and was characterized by plaque formation (24 hpi) and cell lysis between 3–4 days.

### Genome structure and genetic variability of CHIKV isolated in Colombia

**Figure 1:**
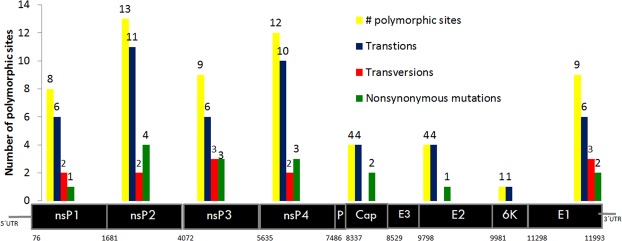
Number of mutations detected in the strains of CHIKV isolated in the present study. Each gene has a range nucleotides position depicted below the genes nsP1 to E1. Each gene shows polymorphic sites (yellow), transitions (blue), transversions (red) and nonsynonymous mutation (green). The Colombian sequences previously published in the Gen-Bank were not taken into account for this analysis.

For each sequence, an average of 35,574,564 paired reads (32,021,178 and 40,904,884) and 3,602,990,602 read bases were obtained. The percentage of GC was 49%. The 16 genomes were assembled with an average length of 12062 (11,394 to 12,347) nucleotides. The average percentage value of the coding regions of the genome (ORF) of CHIKV was 28.8% adenine (A), 25.9% guanine (G), 20.6% thymine (T) and 24.7% cytosine (C). As reported for other CHIKVs, the sequences contained two open reading frames of 7410 nt and 3744 nt that encode the nonstructural polyprotein (2470 aa nsP1-nsP4) and the structural polyprotein (1248 aa E1-E3, 6 K, C), respectively. The length of genes of the strains and the percentage of homology concerns to reference genome LN898093 (Supplementary Table S1). Sixty polymorphic sites were detected among the Colombian sequences (Fig. 1). The 70% (n = 42) of the substitutions occurred in the non-structural proteins and 30% (n = 18) in the structural proteins. We found 8 polymorphic sites in Nsp1 (6 ti + 2 tv), 13 in Nsp2 (11 ti + 2tv), 9 in Nsp3 (6ti + 3tv), 12 in Nsp4 (10ti + 2 tv), 4 in capsid (4 ts), 4 in E2 (4ts), 1 in 6 K (1ts) and 9 in E1 (6 ts + 3 tv). Sixteen out of the 61 polymorphisms were non-synonymous substitutions, and of these, 4 were parsimoniously informative, and 12 were non-informative (Fig. 1).

### Molecular fingerprint and phylogenetic relationship of Colombian CHIKV

**Figure 2:**
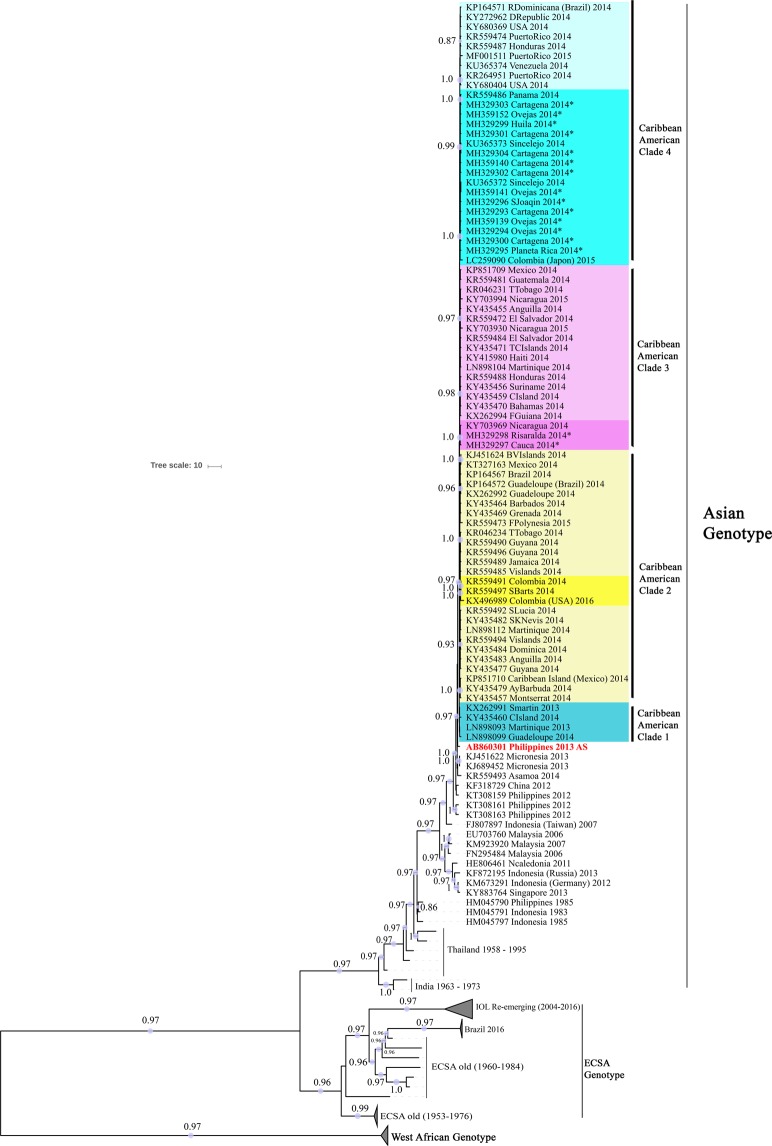
Bayesian inference tree. Taxon labels include access number, country of isolation and year of collection. Subsequent probabilities ≥0.80 are labeled in each branch.

Within the Caribbean American clade (CAC), the Colombian strains were grouped into three subclades with strains of different geographical origins. Each subclade had branch support of value 1 (posterior probability 1), indicating a genuine relationship between grouped strains. i. the Panamanian strain (KR559486-2014) was close to those of the Colombian Caribbean (SJ-Mahates, Cartagena, Ovejas, Planeta Rica, Sincelejo) and Huila; ii. The Nicaraguan strain (KY703969-2014) was close to Risaralda and Cauca; and iii. St. Barts strain (KR559497-2014) was closely related to two Colombian strains, one reported in Bogota and the other one of unknown origin (Fig. 2 and Supplementary Fig. S2).

**Figure 3:**
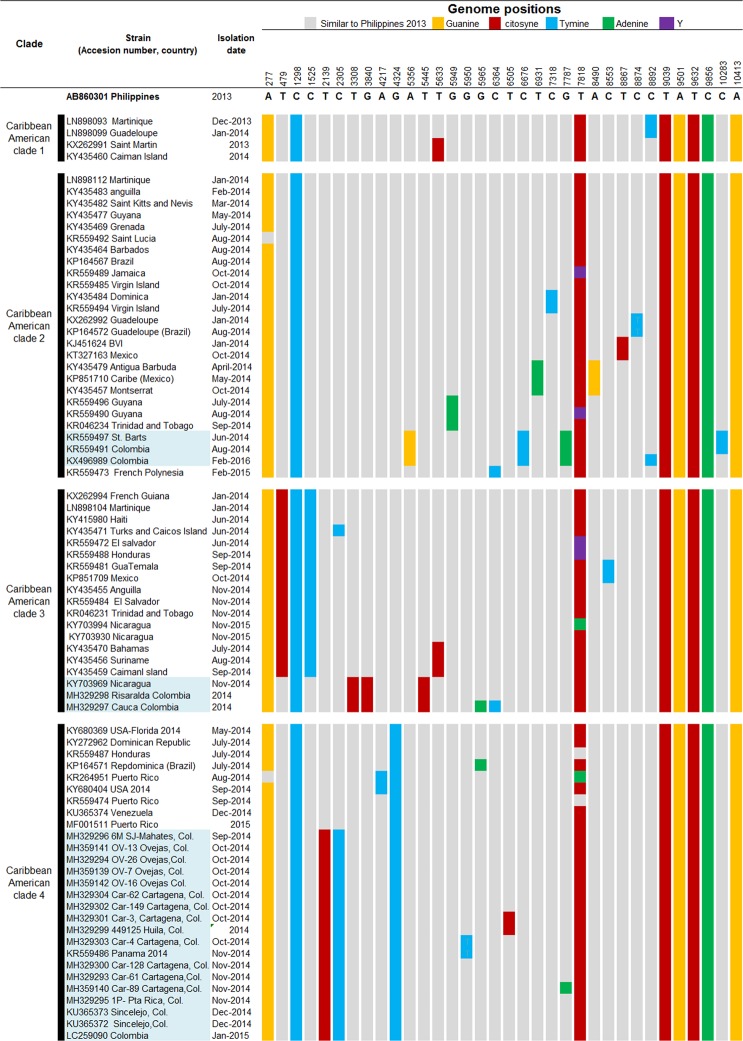
Nucleotide changes that defined subclades within the Caribbean American clade. The gray color represents the nucleotides similar to the Philippine strain AB860301. The yellow color represents a change to guanine, red to cytosine, blue to thymine and green to adenine. Black represents mixed bases within the sequences.

**Figure 4:**
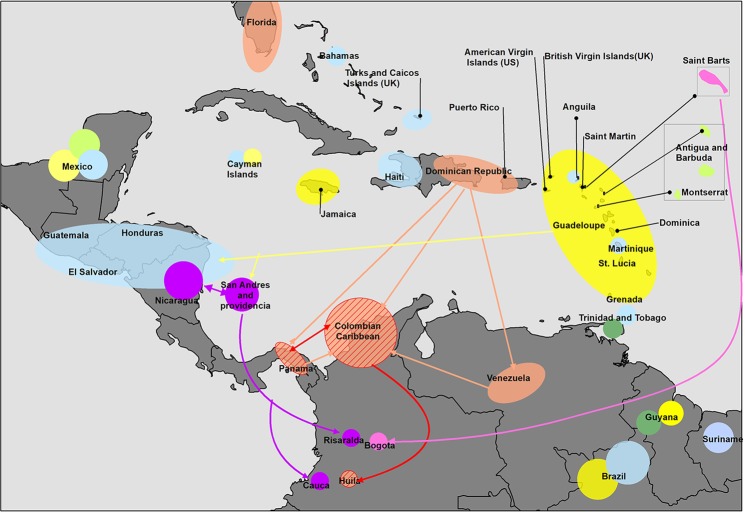
Chikungunya virus spread in the Americas based on the phylogenetic tree and molecular fingerprint. Different colors represent several mutations in Caribbean American clades of CHIKV. Yellow color shows the islands where the initial strains were introduced in the Americas from the Philippines. Pink color shows strains with mutations nsP3-A5356G, nsP4-C6676T and capsid-G7787A (aa. capsid-R78Q); green color (nsP4-G5949A [aa. nsP4-R 99Q]); Orange color (nsP3-G4324T); Orange color with lines (nsP3-G4324T, nsP2-C2305T, nsP2-T2139C [aa. nsP2-A153V]); Blue color (T479C, C1525T); Purple color (nsP2-T3308C [aa. nsp2-Y543H], nsP2-G3840C [aa. nsp2-G720A] and nsP3-T5445C [aa. nsP3-L458P]); green apple color: (nsP4-T6931A, E3-A8490G).

After phylogenetic analysis, polymorphic sites that allowed subclade grouping were found into the strains from America and the Caribbean Islands (Fig. 3). It was observed that the strains of CAC had 8 polymorphic sites; those modifications were different from the ancestral Asiatic strains (Fig. 3). Within the CAC, some polymorphisms allowed differentiation into sub-clades from different countries (Fig. 3). In the case of the Colombian strains and phylogenetically close ones (Nicaragua, Saint Barts, Panamá), it was observed that the mutations T3308C (aa. nsp2-Y543H), G3840C (aa. nsp2-G720A) and T5445C (aa. nsP3-L458P) were only present in the strains of Nicaragua, Risaralda, and Cauca. The mutations nsP3-A5356G, nsP4-C6676T, and capsid-G7787A (aa. capsid-R78Q) were present in 3 isolates from St. Barts, Bogotá and unknown Colombian strain with no geographic description in GenBank. The nsP3-G4324T mutation appeared in the strains of the Dominican Republic, USA (Florida and California), Puerto Rico, Honduras, Venezuela, Panama, the Colombian Caribbean, and Huila. Additionally, T2139C (aa. nsP2-A 153 V) and nsP2-C2305T mutations were detected in the strains of Panama (KR559486), Colombian Caribbean and Huila (Figs 3, 4; Table 1).

**Table 1:** Relevant amino acid substitutions identified in the Colombian CHIKV isolates.

Gene	Nt positión^a^	Shift	Shift AA (gen)^b^	Panama	Car-61	OV26	1 P	6 M	INS-449125	Car-128	Car-3	Car-149	Car-4	Car-62	OV-7	Car-89	OV-13	OV-16	Nicaragua	INS- 477150	INS-449325	Saint Barts	1Colombia	2Colombia
nsP1	398	C > A	L 108 M	.	.	.	.	+	.	.	.	.	.	.	.	.	.	.	.	.	.	.	.	.
nsP2	2139	T > C	A 153 V	+	+	+	+	+	+	+	+	+	+	+	+	+	+	+	.	.	.	.	.	.
nsP2	3308	T > C	Y 543 H	.	.	.	.	.	.	.	.	.	.	.	.	.	.	.	+	+	+	.	.	.
nsP2	3840	G > C	G 720 A	.	.	.	.	.	.	.	.	.	.	.	.	.	.	.	+	+	+	.	.	.
nsP2	4050	T > C	V 790 A	.	+	.	.	.	.	.	.	.	.	.	.	.	.	.	.	.	.	.	.	.
nsP3	5196	G > C	G 375 A	.	+	.	.	.	.	.	.	.	.	.	.	.	.	.	.	.	.	.	.	.
nsP3	5426	C > T	P 452 S	.	.	.	.	.	.	+	.	.	.	.	.	.	.	.	.	.	.	.	.	.
nsP3	5445	T > C	L 458 P	.	.	.	.	.	.	.	.	.	.	.	.	.	.	.	+	+	+	.	.	.
nsP4	5826	C > T	T 58 M	.	.	.	.	.	.	.	.	.	.	.	.	+	.	.	.	.	.	.	.	.
nsP4	6384	G > A	S 244 N	.	.	.	+	.	.	.	.	.	.	.	.	.	.	.	.	.	.	.	.	.
nsP4	6849	C > A	S 399 Y	.	.	.	+	.	.	.	.	.	.	.	.	.	.	.	.	.	.	.	.	.
C	7589	G > A	R 12 K	.	.	.	.	.	.	.	+	.	.	.	.	.	.	.	.	.	.	.	.	.
C	7787	G > A	R 78 Q	.	.	.	.	.	.	.	.	.	.	.	.	+	.	.	.	.	.	+	+	+
E2	8895	T > C	V 373 A	.	.	.	+	.	.	.	.	.	.	.	.	.	.	.	.	.	.	.	.	.
E1	10145	T > C	I 55 T	.	.	+	.	.	.	.	.	.	.	.	.	.	.	.	.	.	.	.	.	.
E1	10283	C > T	T 101 M	.	.	.	.	.	.	.	.	.	.	.	.	.	.	.	.	.	.	+	+	.
E1	11035	T > G	L 352 V	.	.	.	.	.	.	.	.	.	.	.	.	.	.	.	.	.	+	.	.	.

^a^The nucleotide positions are numbered by the Whole genome (including 5′ UTR). ^b^The amino acid positions are numbered from the first amino acid of each gene product.

When we compared the sequences with the closest phylogenetic strain, 40 variable sites were detected between the Panamanian strain (KR559486) and those from SJ-Mahates, Ovejas, Cartagena, Planeta Rica, Sincelejo and Huila (Supplementary Table S3). Of the 40 sites, 37 were unique mutations, and 11 produced changes in the amino acid sequence (Table 1). 72% (n = 29/40) of the substitutions occurred in the non-structural proteins and 28% (n = 11/40) in the structural proteins. We found 5 polymorphic sites in nsP1 (4 ti + 1 tv), 8 in nsP2 (7 ti + 1tv), 7 in nsP3 (5ti + 2tv), 9 in nsP4 (7ti + 2 tv), 4 in capsid (4 ts), 3 in E2 (3ts), and 4 in E1 (3 ts + 1 tv) (Supplementary Table S3). The strains with the most variable sites were those from the Planeta Rica (Córdoba) and Huila, with 12 and 9 polymorphisms, respectively.

When comparing the strains of Risaralda and Cauca with that of Nicaragua (KY703969), 18 unique mutations were detected, one of which produced a change in the amino acid sequence (Table 1 and Supplementary Table S3). 50% (n = 9/18) of the substitutions occurred in the non-structural proteins and 50% (n = 9/18) in the structural proteins. We found 3 polymorphic sites in nsP1 (2 ti + 1 tv), 2 in nsP2 (2ts), 4 in nsP4 (4 ts), 2 in E2 (2ts), 1 in 6 K (1 ts) and 6 in E1 (4 ts + 2 tv) (Supplementary Table S3). The strains of St. Barts and Colombia contained two polymorphisms (2ts + 2tv) of which one produced a change in the amino acid sequence (Table 1 and Supplementary Table S3).

## Discussion

The phylogenetic analysis in this study determined that CHIKVs in Colombia belong to 3 clusters of the Asiatic genotype: Panama (Caribbean Colombia, Huila), Nicaragua (Cauca and Risaralda) and St. Barts (Bogotá, D.C). Our results are similar to previous research that used complete sequencing of worldwide strains and a Bayesian phylogenomics approach^7,23^. In this sense, the previous analysis including three Colombian viral genomes, two from Sincelejo (Sucre) (KU365372.1-KU365373.1) and one from Bogotá D.C, identified a similar phylogenetic relationship between Panama and St. Barts, respectively^7,23^. However, that work focused on a global phylogenic study, and few Colombian strains were analyzed.

On the other hand, our results are different from other studies on phylogenic analyses of Colombian CHIKV. Mattar *et al*.^20^ in 2015 and Rodas *et al*.^24^ in 2016, evaluated Colombian Caribbean strains (Sincelejo, Ovejas, SJ-Mahates) with partial sequences of the E2, 6 K, E1 and nsP1 genes and found a phylogenetic relationship with strains from Virgin Islands, Saint Lucia, Mexico, Puerto Rico, Brazil and China^20,24^. Laiton-Donato *et al*.^14^ in 2015 used the E1 gene to study isolates from 15 departments in Colombia and found that there was a relationship with CHIKV from Mexico, Brazil, Virgin Islands, and Saint Martin^14^. Other recent studies^25^ in Colombia evaluated strains of Norte de Santander and found a relationship with the Colombian Caribbean strains and China^25^. It should be noted that the previous phylogenetic analyses carried out in Colombia included strains of few countries and partial sequences of genes, thus precluding a complete evaluation of dynamic evolution and phylogenetic relationships of studied strains^21^. In contrast, our research used whole viral genomes and 157 isolates from affected countries, allowing us to identify a significant number of informative sites, reflecting a virus evolutionary history.

When analyzing the polymorphisms, the mutations nsP2-C-2305-T and nsP2-C-2139 and (AA, nsP2-V153A) confirmed the phylogenetic relationship between the isolates of Panama, the Colombian Caribbean, and Huila (Figs 3, 4). The mutation nsP3 G-4324-T suggested that the strains of Panama, Colombian Caribbean, and Huila could come from Venezuela, the United States, Puerto Rico, the Dominican Republic or Honduras (Figs 3, 4). Of the countries that make up this clade, the Dominican Republic was the first country to report cases of indigenous transmission of CHIKV (February 2014)^26^ followed by Puerto Rico (May 2014)^3^, USA, Venezuela, Brazil, Panama (July 2014)^3,22^ and Colombia (September 2014)^3,27^. With the information systems of migratory passenger mobility and the surveillance system in Public Health of Colombia, it was possible to establish that during May and July 2014, infected travelers from the Dominican Republic arrived in Panama and Colombia respectively (Epidemiological Bulletin Colombia)^19,28^. This epidemiological information suggests that the route of introduction to the Colombian Caribbean was through a strain that was circulating previously on the Dominican Republic and later was introduced to Panamá, Colombian Caribbean, and Huila.

On May 13, 2014, Panama reported its first imported cases passengers coming from the Dominican Republic and Haiti^29^. Colombia’s first imported cases came from Venezuela, the Dominican Republic, and Panama and were reported more than two months later, in late July^19^. The high migration of travelers between Panama and Colombia facilitates the transmission of diseases between these countries; according to the Ministry of Foreign Affairs of Colombia, in July 2014, there were 34,413 travelers between Panama and Colombia^28^. For the same period, Cartagena was the Colombian city with the third highest number of foreigners (20,800 people)^28^. Therefore, it is possible that the Panamanian strain of CHIKV has entered through Cartagena (Bolivar) and not through SJ-Mahates (50 km away from Cartagena), where the first autochthonous cases were detected^27^.

In interviews carried out with the habitants of SJ-Mahates and its surrounding rural areas, it was established that infected people from Venezuela and Cartagena preceded the time of the outbreak in SJ-Mahates^27^. The first cases in Cartagena were related to those cases arriving from Venezuela and the Dominican Republic^27^. This epidemiological information also allows us to suggest that the introduction of CHIKV to the Colombian Caribbean may have come from Venezuela and the Dominican Republic and the additional mutations nsP2 C-2305-T, T-2139-C (aa.ns P2-V153A) arose in Colombia and were imported into Panama.

In the last 5 to 10 years, Venezuela has faced a severe economic crisis, precipitated by political instability^30^. Public health has been affected, and the country is experiencing an increase and expansion of vector-borne diseases^30^. The United Nations High Commissioner for Refugees estimates that between 2014 and March 2018, around 600,000 people sought refuge in Colombia, not counting the people who migrated through illegal border crossings^30^. Despite the migration of Venezuelans to Colombia and all the cases of CHIKV that were imported from Venezuela to Colombia before and during the epidemic^27^, in this study no close phylogenetic relationship was found with Venezuelan strains. However, the circulation of these strains in Colombian border areas with Venezuela, such as Guajira and Norte de Santander cannot be ruled out. In this sense, the possibility for detecting Venezuelan or other countries’ strains in Colombia was affected by the limited number of sequences from some territories of the Americas and the absence of samples from other areas of Colombia.

Concerning the strains found in the Risaralda and Cauca Andean area, the T3308C (aa. nsp2-Y543H), G3840C (aa. nsp2-G720A) and T5445C (aa. nsP3-L458P) mutations demonstrate their phylogenetic relationship with the Nicaraguan strain (Figs 3, 4). In a previous study, it was determined that the Nicaraguan strain was closely related to the strain of British Virgin Islands^31^. In contrast, our study showed that strain BVI KJ451624 has a relationship to Mexican strain (Fig. 2). BVI KJ451624 strain was one of the first to appear in the outbreak in January 2014 in the Caribbean islands; it is possible that the strains of CHIKV mutated in their movement from Sant Martin December 2013, British Virgin Islands, Mexico, Nicaragua, and Colombia, where the mutations were detected.

It is difficult to determine how the Nicaraguan strain reached the South West of the Andean region of the country (Risaralda and Cauca). One possibility is that the strain entered from Nicaragua through the Island of San Andres since the border is only 233 km away and San Andres is one of the most visited tourist sites by people of the Andean area of Colombia (Fig. 4). However, it could also be in the opposite direction, so that the strains of San Andres islands migrated to Nicaragua. Unfortunately, in this study, it was not possible to include strains of San Andres thus not allowing us to make inferences about this phenomenon of viral circulation.

Regarding the strain Saint Barts 2014, the phylogenetic analysis demonstrated a close relationship with two Colombian strains of 2014 (KR559491) and 2016 (KX496989) (Figs 2, 3). The strain KX496989 was isolated from an American woman who traveled to Bogotá D.C and had a co-infection with Zika virus and CHIKV^32^. The altitude of Bogotá at 2600 meters above sea level, does not support the populations of the *Aedes aegypti* vector^33^; however, is possible that the woman was infected in another municipality of Cundinamarca, since in the description of the clinical case it is indicated that the woman visited urban and rural areas where she received mosquito bites^32^. According to the reports of the National Institute of Health, between the years 2014–2016 in Cundinamarca, there were 19,919 cases of CHIKV^17,18^. In this sense, our study suggests that the CHIKV of Saint Barts circulates in Cundinamarca or nearby departments.

The detection of polymorphisms A5356G, C6676T and G7787A (aa.C-R78Q) in strains isolated more than two years apart (2014 to 2016), suggests that the CHIKV of Saint Bart and Colombia is a variant within the Caribbean American clade (Fig. 3). Interestingly, the G7787A mutation (aa Capside-R78Q) was also detected in the car-89 strain of Cartagena (Table 1). This finding in strain Car-89 may have been the result of an error-matched viral RNA polymerase, or due to a recombination event, which has already been reported for CHIKV and other alphaviruses. In this study, we have no certainty of what could have happened, and neither were analyzes performed to detect possible recombinant events. It would be interesting to investigate if this variant still circulates in Colombia and if additional mutations have occurred from 2016 to 2019.

In addition to the mutations that were identified in each clade, we found that the Colombian strains had 60 polymorphic sites, of which 70% (n = 42/60) occurred in non-structural proteins and 30% (n = 18) in structural proteins (Table 1 and supplementary Table S3). These findings are similar to those reported by Stapleford *et al*.^34^, who through deep sequencing searched for the frequency of viral minority populations in the same individual and found that the majority of CHIKV polymorphisms occurred in non-structural proteins and 3′UTR^34^.

The biological function of synonymous and non-synonymous substitutions in Colombian CHIKV is unknown, and it would be interesting to carry out future studies to understand how these changes in the genome influence the pathogenesis and transmission of CHIKV^35,36^. Of the adaptive mutations in E1 and E2 previously reported, the Colombian strains contain the mutations E2-G60D and E2-I211T^37^. It has been proved that E2-G60D increases the infectivity in *A*. *albopictus* and *A*. *aegypti*, whatever the amino acid (alanine or valine) in position E1-22636^36^. On the other hand, E2-I211T increases the infectivity in *Ae*. *albopictus* of strains CHIKV E1-226V, but does not affect *Ae*. *Aegypti*^37,38^. It is important to note that Colombian strains, as well as other Asian strains, contain threonine in position 98 of E1 (E1-98T), which limits their ability to infect *Ae*. *albopictus* through the substitution E1-A226V^37^.

In conclusion, the genetic diversity and polymorphism in several American countries of CHIKV genomes implied evolutionary diversification and possible adaptation to human ecosystems and insect vectors. The subclades showed different non-synonymous mutations with likely functional consequences for CHIKV biology and pathogenesis.

## Methods

### Ethical approval and informed consent

Institutional standard guidelines of the Minister of Health of Colombia and the University of Cordoba ethics committee were followed for the collection of patients’ blood samples after their written informed consent for involvement in the study was obtained. Sera of pediatric patients from Hospital Infantil Napoleon Franco, Cartagena, Colombia were authorized by the ethics committee of the Hospital (Acta # 6 October 15th, 2015) and informed consent was obtained from all patients under the authorization of their parents. All patients included in the study were under an anonymous code number. The study incorporated procedures, management, and conservation of samples, and technical-administrative procedures for health research required by resolution 8430 of the Ministry of Health of Colombia, in 1993^39^, and declaration of Helsinki for ethical and medical research in human subjects^40^.

### Specimens

**Figure 5:**
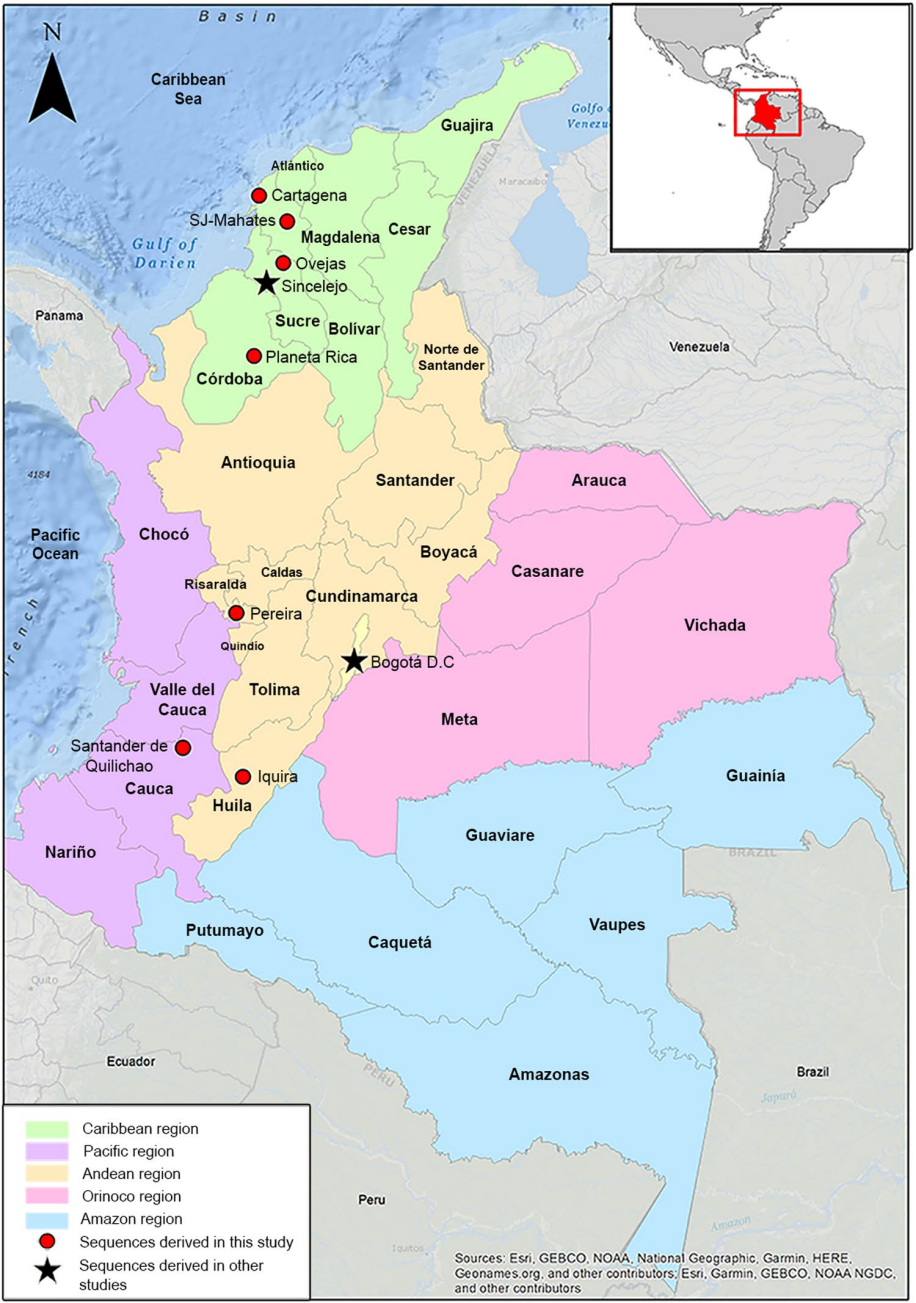
Map of Colombia showing the isolation sites of the strains studied.

Between September and December 2014 during the epidemic outbreak in the Caribbean area of Colombia, a collection of 57 sera was selected^20,41^. Thirty-six sera were from Cartagena (Department of Bolivar), 19 from Ovejas (Department of Sucre), 1 from San Joaquin-Mahates (Department of Bolivar) and 1 from Planeta Rica (Department of Córdoba). The specimens came from pediatric and adult patients, had complete clinical and epidemiological records and were positive for CHIKV by qRT-PCR (LightMix Chikungunya Virus Kit, Roche, USA). The viral loads ranged between 1.06 × 10^1^–1.25 × 10^7^ copies/mL. The National Institute of Health of Colombia (INS) donated three strains isolated from the Departments of Risaralda, Huila, and Cauca, all of them belonging to the Andean area of Colombia (Fig. 5).

### Cell culture and viral isolation

The 57 sera were cultured onto C6/36 and Vero cells, maintained in 12.5 cm2 flasks, in Leibovitz 15 and DMEM (Dulbecco’s Modified Eagle Medium) media respectively. Media were supplemented with 10% fetal bovine serum (FBS), 2 mM L-glutamine, 1X non-essential amino acids, 100 units/ml penicillin, 100 µg/ml streptomycin and 2 µg amphotericin B. C6/36 cells were incubated at 28 °C and Vero cells 37 °C under 5% CO2. For viral isolation, 40 µl of serum were added to confluent monolayers of C6/36 or Vero cells and incubated for 1 hour at 28 °C and 37 °C respectively. After viral adsorption, the sample was removed, and 3 ml of medium supplemented with 2% FBS was added and incubated at 28 °C or 37 °C according to the cell line. At the end of 14 day or following early recognition of the cytopathic effect (CPE), the cellular supernatants were collected and stored at −80 °C. All the cell culture procedures were carried out at Biological Research Institute of the Tropic of the University of Cordoba with equipment’s and efficient practices of a level of biosafety II, that involve infectious or potentially infectious material.

### RNA extraction, RT-PCR, and confirmation of CHIKV

The RNA was obtained from 140 μl of the supernatant of the cell cultures using the QIAamp Viral RNA Mini Kit (Qiagen, France), following the manufacturer’s recommendations. For the synthesis of cDNA, M-MLV Reverse Transcriptase (200 U/μl, Promega) and Random Primers (0.5 μg/μl, Promega) were used. For the confirmation of CHIKV in viral cultures, TaqMan Fast Universal PCR Master Mix (2X) (Applied Biosystems) was used. For each reaction, 10 µl of 2X buffer were added; 0.18 µl of forward primer (final concentration 0.9 µM); 0.18 µl of reverse primer (final concentration 0.9 μM); 0.1 µl of probe (final concentration 0.25 μM); 4.54 µl of RNase-free water and 5 µl of cDNA. As positive controls, a culture of CHIKV confirmed by conventional PCR was used; ultra-pure water served as a negative control. Samples with a Ct less than or equal to 38 were considered positive

### Amplification of the CHIKV genome by PCR

16 viral isolates of CHIKV were selected based on their geographical origin: Cartagena n = 7, San Joaquin-Mahates = 1, (Department of Bolivar), Ovejas n = 4 (Department of Sucre), Planeta Rica n = 1 (Department of Cordoba), Department of Risaralda n = 1, Department of Cauca n = 1, Department of Huila n = 1 (Table 2). For amplification of the CHIKV genome, twelve sets of primers previously reported by Stapleford *et al*.^34^ with modifications, were used (Supplementary Table S4). The primers amplified overlapping regions of the retrotranscribed genomes. The PCR reaction was carried out with the Exact Taq PLUS 2X Mastermix. The amplified products were verified by electrophoresis in 1% agarose gel using SYBR safe DNA Gel Stain. The amplicons of each sample were mixed in equimolar concentration, to avoid unequal distribution of data in the steps of library preparation and sequencing.

**Table 2:** Colombian strains, patient’s information and sequenced genome of the present study.

Sample	Strain	GenBank accession number	Year	Gender	Age	City	Days of evolution	Viral load (copies /ml) (serum)
1	Car-61	MH329293	2014	F	2 (months)	Cartagena	0	2.52 × 10^4^
2	OV-26	MH329294	2014	F	71 (years)	Ovejas	2	2,50 × 10^6^
3	1 P	MH329295	2014	M	33 (years)	Planeta Rica	3	3,60 × 10^2^
5	6 M	MH329296	2014	F	32 (years)	San Joaquín-Mahates	3	7,40 × 10^2^
19	INS- 477150	MH329297	2014	NA	NA	Cauca	NA	NA
20	INS-449325	MH329298	2014	NA	NA	Risaralda	NA	NA
21	INS-449125	MH329299	2014	NA	NA	Huila	NA	NA
36	Car-128	MH329300	2014	F	6 (days)	Cartagena	1	3.44 × 10^4^
37	Car-3	MH329301	2014	F	16 (days)	Cartagena	3	1.72 × 10^2^
38	Car-149	MH329302	2014	M	5 (days)	Cartagena	NA	1.37 × 10^6^
39	Car-4	MH329303	2014	F	1 (months)	Cartagena	3	1.53 × 10^6^
40	Car-62	MH329304	2014	M	10 (days)	Cartagena	2	1.57 × 10^6^
41	OV-7	MH359139	2014	F	12 (years)	Ovejas	2	1,70 × 10^5^
42	Car-89	MH359140	2014	M	1 (months)	Cartagena	3	2.00 × 10^4^
43	OV-13	MH359141	2014	F	27 (years)	Ovejas	1	6,20 × 10^5^
44	OV-16	MH359142	2014	F	19 (years)	Ovejas	3	6,20 × +10^3^

NA = not available.

### Sequencing and assembly of genomes

The libraries were prepared using a TruSeq DNA Sample Prep Kit (Illumina). Generation of clusters and fragment sequencing was performed using a Truseq SBS Kit v4 and Illumina HiSeq 2500 (2-x 101pb paired-end). The fastq files were processed using Trimmomatic v0.36^42^, for the removal of low-quality sequences. The genomes were assembled with the Iterative Virus Assembler software and the reference strain LN898093 of Martinique^43^.

### Preparation of data

A first dataset (CHIKV3gen; n = 173) was generated with the 16 sequences assembled in this study and 157 complete genomes of CHIKV available in GenBank. The GenBank sequences belonged to the three genotypes, with collection dates between the years 1953–2016 (Supplementary Table S5). The 173 sequences were aligned with MUSCLE v3.8.31^44^ and manually curated while retaining the codon homology with the Geneious v11.1.2 program^45^. A second dataset (CHIKVas; n = 104) was prepared from CHIKV3gen and contained only Asian CHIKVs from 1953–2016. Due to the ambiguous alignment of the UTR, only the open reading frames (ORFs) comprising 11256 nucleotides were used for the reconstruction of the phylogenetic tree and subsequent analyses. The nucleotide and amino acid positions used were based on the reference sequences KX262995, KY055011 and LN898093. All sequences were identified by the date of sampling and the location from which they originated.

### Phylogenetic reconstruction

The Bayesian phylogenetic inference of the ORF alignments was made using a molecular and uncorrelated clock with the BEAST v2 program^46^. The analysis was run in duplicate with 90 million replicates, and the trees were sampled every 1000 steps. The coalescence model implemented was a Bayesian Skyline prior with a relaxed clock model and variation rates between branches using a log-normal distribution and the substitution model recommended by jModel Test (GTR + G + I). The convergence of MCMC chains was verified with Tracer v1. 7. The trees obtained were edited using the software FigTree v1.4.3. The alignment of the second dataset CHIKVas; (n = 104) which contained Asian genotypes was used to determine the genetic diversity (Software MEGA 7).

## Supplementary information

Supporting information
