# Supplementary material for: Genomic epidemiology of Chikungunya virus in Colombia reveals genetic variability of strains and multiple geographic introductions in outbreak, 2014
Source: Sci Rep. 2019 Jul 10;9:9970. doi: 10.1038/s41598-019-45981-8 (PMC6620336; doi:10.1038/s41598-019-45981-8)
Supplement: Supplementary file 1 — Supporting information [file 41598_2019_45981_MOESM1_ESM.pdf]

Genomic epidemiology of *Chikungunya* virus in Colombia reveals genetic variability of strains and multiple geographic introductions in outbreak, 2014

Yeneiris Villero-Wolf[1], Salim Mattar[1,2*], Andrés Puerta-González[3], German Arrieta[1,4], Carlos Muskus[5], Richard Hoyos[6], Hernando Pinzon[7], Dioselina Peláez-Carvajal[8] .

[1] Instituto de Investigaciones Biológicas del Trópico, Universidad de Córdoba, Montería, Córdoba, Colombia.

[2] Clínica Salud Social, Sincelejo, Sucre, Colombia.

[3]Universidad de Antioquia, Medellín, Antioquia, Colombia.

[4]Grupo de Salud Pública, Corporación Universitaria del Caribe-CECAR, Sincelejo, Sucre, Colombia.

[5]Programa de Estudio y Control de Enfermedades Tropicales (PECET), Facultad de Medicina, Universidad de Antioquia, Medellín, Antioquia, Colombia.

[6]Grupo de Investigación en Resistencia Bacteriana y Enfermedades Tropicales, Universidad del Sinú, Montería, Córdoba, Colombia.

[7]Universidad de Cartagena, Hospital Infantil Napoleon Franco, Cartagena, Colombia.

[8]Grupo de virología, Instituto Nacional de Salud. Bogotá DC, Colombia.

Supplementary Table S1. Description of the genomes sequenced. Lengths of each gene of each strain and percentage of homology is concerning to reference genome LN898093.

		Non-structural polyproteins					structural polyproteins				
**Strain**	**Lenght**	**NSP1 (% of reference)**	**NSP2 (% of reference)**	**NSP3 (% of reference)**	**NSP4 (% of reference)**	**Pro (% of reference)**	**C (% of reference)**	**E3 (% of reference)**	**E2 (% of reference)**	**6K (% of reference)**	**E1 (% of reference)**
CAR-61	11886	50-1654 (99.938)	1655-4045 (99.875)	4046-5626 (99.873)	5627-7459 (100)	7463-7527 (100)	7528-8310 (100)	8311-8502 (100)	8503-9771 (99.921)	9772-9954 (100)	9955-11271 (100)
INS- 477150	12185	62-1666 (99.938)	1667-4057 (99.875)	4058-5638 (99.937)	5639-7471 (99.836)	7475-7539 (100)	7540-8322 (100)	8323-8514 (100)	8515-9783 (99.842)	9784-9966 (100)	9967-11283 (99.772)
OV-26	11709	61-1665 (100)	1666-4056 (99.916)	4057-5637 (99.937)	5638-7470 (100)	7474-7538 (100)	7539-8321 (100)	8322-8513 (100)	8514-9782 (99.921)	9783-9965 (100)	9966-11282 (99.924)
INS-449325	12183	61-1665 (99.875)	1666-4056 (99.875)	4057-5637 (99.937)	5638-7470 (99.945)	7474-7538 (100)	7539-8321 (100)	8322-8513 (100)	8514-9782 (99.921)	9783-9965 (99.454)	9966-11282 (99.772)
INS-449125	12128	62-1666 (99.875)	1667-4057 (99.916)	4058-5638 (99.810)	5639-7471 (99.891)	7475-7539 (100)	7540-8322 (99.872)	8323-8514 (100)	8515-9783 (99.921)	9784-9966 (100)	9967-11283 (100)
P1	11959	61-1665 (100)	1666-4056 (99.833)	4057-5637 (99.810)	5638-7470 (99.891)	7474-7538 (100)	7539-8321 (100)	8322-8513 (100)	8514-9782 (99.685)	9783-9965 (100)	9966-11282 (99.924)
CAR-128	12043	61-1665 (99.938)	1666-4056 (99.833)	4057-5637 (99.873)	5638-7470 (100)	7474-7538 (100)	7539-8321 (100)	8322-8513 (100)	8514-9782 (99.921)	9783-9965 (100)	9966-11282 (100)
CAR-3	12347	61-1665 (100)	1666-4056 (99.916)	4057-5637 (99.937)	5638-7470 (99.945)	7474-7538 (100)	7539-8321 (99.872)	8322-8513 (100)	8514-9782 (99.921)	9783-9965 (100)	9966-11282 (100)
CAR-149	12306	61-1665 (100)	1666-4056 (99.916)	4057-5637 (99.937)	5638-7470 (100)	7474-7538 (100)	7539-8321 (100)	8322-8513 (100)	8514-9782 (99.921)	9783-9965 (100)	9966-11282 (100)
CAR-4	11694	61-1665 (100)	1666-4056 (99.916)	4057-5637 (99.873)	5638-7470 (99.945)	7474-7538 (100)	7539-8321 (100)	8322-8513 (100)	8514-9782 (99.921)	9783-9965 (100)	9966-11282 (100)
CAR-62	12270	61-1665 (100)	1666-4056 (99.916)	4057-5637 (99.937)	5638-7470 (100)	7474-7538 (100)	7539-8321 (100)	8322-8513 (100)	8514-9782 (99.921)	9783-9965 (100)	9966-11282 (100)
7 OV	12153	61-1665 (100)	1666-4056 (99.916)	4057-5637 (99.937)	5638-7470 (99.891)	7474-7538 (100)	7539-8321 (99.872)	8322-8513 (100)	8514-9782 (99.921)	9783-9965 (100)	9966-11282 (99.924)
CAR-89	12191	61-1665 (100)	1666-4056 (99.875)	4057-5637 (99.937)	5638-7470 (99.945)	7474-7538 (100)	7539-8321 (99.872)	8322-8513 (100)	8514-9782 (99.921)	9783-9965 (100)	9966-11282 (100)
OV-13	12086	62-1666 (100)	1667-4057 (99.916)	4058-5638 (99.937)	5639-7471 (100)	7475-7539 (100)	7540-8322 (100)	8323-8514 (100)	8515-9783 (99.921)	9784-9966 (100)	9967-11283 (100)
0V-16	12160	61-1665 (100)	1666-4056 (99.875)	4057-5637 (99.937)	5638-7470 (100)	7474-7538 (100)	7539-8321 (100)	8322-8513 (100)	8514-9782 (99.921)	9783-9965 (100)	9966-11282 (100)
M6	11694	74-1678 (99.938)	1679-4069 (99.916)	4070-5650 (99.937)	5651-7483 (100)	7487-7551 (100)	7552-8334 (100)	8335-8526 (100)	8527-9795 (99.921)	9796-9978 (100)	9979-11295 (100)

Supplementary Figure S2. Bayesian inference tree represented as cladogram. Taxon labels include access number, country of isolation and year of collection. Subsequent probabilities ≥0.80 are labeled in each branch.

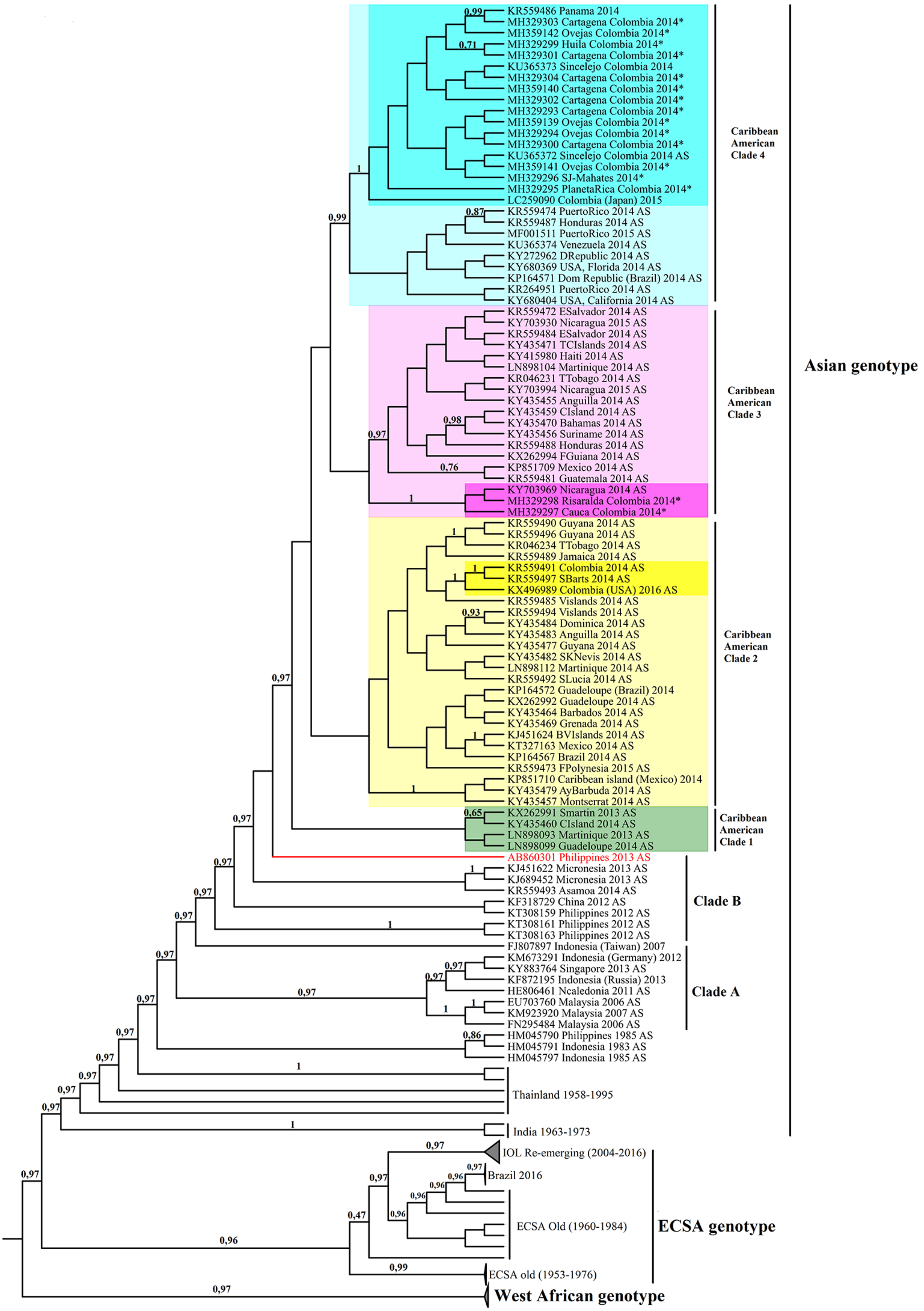

Supplementary Table S3. **Detected polymorphisms and phylogenetic relationship of Colombian strains.** Non-synonymous and synonymous changes that distinguish CHIKV sequences from Colombia from other closely related countries (Panama, Nicaragua, St. Barts).

Positions

Strain	City	nsP1								nsP2													
		337	398	646	689	859	1219	1342	1453	1799	1810	1936	2139	2185	2263	2299	2305	3308	3364	3436	3589	3840	4050
KX262991	Saint Martin	A	C	T	C	A	C	A	G	C	T	A	T	G	C	G	C	T	C	C	G	G	T
KR559486	Panamá	.	.	.	.	.	.	.	.	.	.	.	**C**	.	.	.	T	.	.	.	.	.	.
KU365372	Sincelejo	.	.	.	.	.	.	.	.	.	.	.	**C**	.	.	.	T	.	.	.	.	.	.
KU365373	Sincelejo	.	.	.	.	.	.	.	.	.	.	.	**C**	.	.	.	T	.	.	.	.	.	.
LC259090	Colombia	.	.	.	.	.	.	.	.	T	.	.	**C**	.	.	.	T	.	.	.	.	.	.
Car-61	Cartagena	.	.	C	.	.	.	.	.	.	.	.	**C**	.	.	.	T	.	.	.	.	.	**C**
OV-26	Ovejas	.	.	.	.	.	.	.	.	.	.	.	**C**	.	.	.	T	.	.	.	.	.	.
1P	Pta Rica	.	.	.	.	.	.	.	.	.	.	.	**C**	.	.	.	T	.	T	T	.	.	.
6M	SJ-Mahates	.	**A**	.	.	.	.	.	.	.	.	.	**C**	.	.	.	T	.	.	.	.	.	.
INS-449125	Huila	.	.	.	T	.	T	.	.	.	.	.	**C**	.	.	.	T	.	.	.	.	.	.
Car-128	Cartagena	.	.	.	.	.	.	G	.	.	.	.	**C**	A	.	.	T	.	.	.	A	.	.
Car-3	Cartagena	.	.	.	.	.	.	.	.	.	.	.	**C**	.	.	.	T	.	.	.	.	.	.
Car-149	Cartagena	.	.	.	.	.	.	.	.	.	.	.	**C**	.	.	.	T	.	.	.	.	.	.
Car-4	Cartagena	.	.	.	.	.	.	.	.	.	.	.	**C**	.	.	.	T	.	.	.	.	.	.
Car-62	Cartagena	.	.	.	.	.	.	.	.	.	.	.	**C**	.	.	.	T	.	.	.	.	.	.
OV-7	Ovejas	.	.	.	.	.	.	.	.	.	.	.	**C**	.	.	.	T	.	.	.	.	.	.
Car-89	Cartagena	.	.	.	.	.	.	.	.	.	A	.	**C**	.	.	.	T	.	.	.	.	.	.
OV-13	Ovejas	.	.	.	.	.	.	.	.	.	.	.	**C**	.	.	.	T	.	.	.	.	.	.
OV-16	Ovejas	.	.	.	.	.	.	.	.	.	.	.	**C**	.	T	.	T	.	.	.	.	.	.
KY703969	Nicaragua	.	.	.	.	.	.	.	.	.	.	.	.	.	.	.	.	**C**	.	.	.	**C**	.
INS- 477150	Cauca	.	.	.	.	.	.	.	T	.	.	.	.	.	.	A	.	**C**	.	.	.	**C**	.
INS-449325	Risaralda	G	.	.	.	G	.	.	.	.	.	G	.	.	.	.	.	**C**	.	.	.	**C**	.
KR559497	St. Barts	.	.	.	.	.	.	.	.	.	.	.	.	.	.	.	.	.	.	.	.	.	.
KR559491	Colombia	.	.	.	.	.	.	.	.	.	.	.	.	.	.	.	.	.	.	.	.	.	.
KX496989	Colombia	.	.	.	.	.	.	.	.	.	.	.	.	.	.	.	.	.	.	.	.	.	.

Strain	City	nsP3										nsP4													
		4324	4396	4408	4687	5176	5196	5263	5356	5426	5445	5826	5950	5965	5986	6364	6384	6505	6559	6649	6676	6736	6823	6849	7435
KX262991	Saint Martin	G	T	A	A	A	G	G	A	C	T	C	G	G	C	C	G	T	T	G	C	T	G	C	C
KR559486	Panamá	T	.	.	.	.	.	.	.	.	.	.	T	.	.	.	.	.	.	.	.	.	A	.	.
KU365372	Sincelejo	T	.	.	.	.	.	.	.	.	.	.	.	.	.	.	.	.	.	.	.	.	.	.	.
KU365373	Sincelejo	T	.	.	.	.	.	.	.	.	.	.	.	.	.	.	.	.	.	.	.	.	.	.	.
LC259090	Colombia	T	.	.	.	.	.	.	.	.	.	.	.	.	.	.	.	.	.	.	.	.	.	.	.
Car-61	Cartagena	T	.	.	.	.	**C**	.	.	.	.	.	.	.	.	.	.	.	.	.	.	.	.	.	.
OV-26	Ovejas	T	.	.	.	.	.	.	.	.	.	.	.	.	.	.	.	.	.	.	.	.	.	.	.
1P	Pta Rica	T	.	.	.	G	.	A	.	.	.	.	.	.	.	.	**A**	.	.	.	.	.	.	**A**	.
6M	SJ-Mahates	T	.	.	.	.	.	.	.	.	.	.	.	.	.	.	.	.	.	.	.	.	.	.	.
INS-449125	Huila	T	C	.	G	.	.	.	.	.	.	.	.	.	.	.	.	C	.	.	.	C	.	.	.
Car-128	Cartagena	T	.	.	.	.	.	.	.	**T**	.	.	.	.	.	.	.	.	.	.	.	.	.	.	.
Car-3	Cartagena	T	.	.	.	.	.	.	.	.	.	.	.	.	.	.	.	C	.	.	.	.	.	.	.
Car-149	Cartagena	T	.	.	.	.	.	.	.	.	.	.	.	.	.	.	.	.	.	.	.	.	.	.	.
Car-4	Cartagena	T	.	T	.	.	.	.	.	.	.	.	T	.	.	.	.	.	.	.	.	.	.	.	.
Car-62	Cartagena	T	.	.	.	.	.	.	.	.	.	.	.	.	.	.	.	.	.	.	.	.	.	.	.
OV-7	Ovejas	T	.	.	.	.	.	.	.	.	.	.	.	.	T	.	.	.	.	.	.	.	.	.	T
Car-89	Cartagena	T	.	.	.	.	.	.	.	.	.	**T**	.	.	.	.	.	.	.	.	.	.	.	.	.
OV-13	Ovejas	T	.	.	.	.	.	.	.	.	.	.	.	.	.	.	.	.	.	.	.	.	.	.	.
OV-16	Ovejas	T	.	.	.	.	.	.	.	.	.	.	.	.	.	.	.	.	.	.	.	.	.	.	.
KY703969	Nicaragua	.	.	.	.	.	.	.	.	.	**C**	.	.	.	.	.	.	.	.	.	.	.	.	.	.
INS- 477150	Cauca	.	.	.	.	.	.	.	.	.	**C**	.	.	A	.	T	.	.	C	.	.	.	.	.	.
INS-449325	Risaralda	.	.	.	.	.	.	.	.	.	**C**	.	.	.	.	.	.	.	.	A	.	.	.	.	.
KR559497	St. Barts	.	.	.	.	.	.	.	G	.	.	.	.	.	.	.	.	.	.	.	T	.	.	.	.
KR559491	Colombia	.	.	.	.	.	.	.	G	.	.	.	.	.	.	.	.	.	.	.	T	.	.	.	.
KX496989	Colombia	.	.	.	.	.	.	.	G	.	.	.	.	.	.	.	.	.	.	.	T	.	.	.	.

Strain	City	C				E2						6K	E1										
		7589	7602	7787	7893	8892	8895	9067	9183	9558	9647	9847	10134	10145	10215	10283	10419	10581	10815	11001	11035	11181	11250
KX262991	Saint Martin	G	T	G	C	C	T	T	T	C	T	T	T	T	T	C	T	T	C	G	T	A	T
KR559486	Panamá	.	.	.	.	.	.	.	.	.	.	.	.	.	.	.	.	.	.	.	.	.	.
KU365372	Sincelejo	.	.	.	.	.	.	.	.	.	.	.	.	.	.	.	.	.	.	.	.	.	.
KU365373	Sincelejo	.	.	.	.	.	.	.	.	.	.	.	.	.	.	.	.	.	.	.	.	.	.
LC259090	Colombia	.	.	.	.	.	.	.	.	.	.	.	.	.	.	.	.	.	.	.	.	G	.
Car-61	Cartagena	.	.	.	.	.	.	.	.	.	.	.	.	.	.	.	.	.	.	.	.	.	.
OV-26	Ovejas	.	.	.	.	.	.	.	.	.	.	.	.	**C**	.	.	.	.	.	.	.	.	.
1P	Pta Rica	.	.	.	.	.	**C**	.	.	T	C	.	.	.	.	.	A	.	.	.	.	.	.
6M	SJ-Mahates	.	.	.	.	.	.	.	.	.	.	.	.	.	.	.	.	.	.	.	.	.	.
INS-449125	Huila	.	.	.	T	.	.	.	.	.	.	.	.	.	.	.	.	.	.	.	.	.	.
Car-128	Cartagena	.	.	.	.	.	.	.	.	.	.	.	.	.	.	.	.	.	.	.	.	.	.
Car-3	Cartagena	**A**	.	.	.	.	.	.	.	.	.	.	.	.	.	.	.	.	.	.	.	.	.
Car-149	Cartagena	.	.	.	.	.	.	.	.	.	.	.	.	.	.	.	.	.	.	.	.	.	.
Car-4	Cartagena	.	.	.	.	.	.	.	.	.	.	.	.	.	.	.	.	.	.	.	.	.	.
Car-62	Cartagena	.	.	.	.	.	.	.	.	.	.	.	.	.	.	.	.	.	.	.	.	.	.
OV-7	Ovejas	.	C	.	.	.	.	.	.	.	.	.	.	.	.	.	.	.	T	.	.	.	.
Car-89	Cartagena	.	.	**A**	.	.	.	.	.	.	.	.	.	.	.	.	.	.	.	.	.	.	.
OV-13	Ovejas	.	.	.	.	.	.	.	.	.	.	.	.	.	.	.	.	.	.	.	.	.	.
OV-16	Ovejas	.	.	.	.	.	.	.	.	.	.	.	.	.	.	.	.	.	.	.	.	.	.
KY703969	Nicaragua	.	.	.	.	.	.	.	C	.	.	.	.	.	.	.	.	.	.	.	.	.	.
INS- 477150	Cauca	.	.	.	.	.	.	C	.	.	.	.	C	.	C	.	.	.	.	C	.	.	.
INS-449325	Risaralda	.	.	.	.	.	.	.	.	.	.	C	.	.	.	.	.	C	.	.	**G**	.	C
KR559497	St. Barts	.	.	**A**	.	.	.	.	.	.	.	.	.	.	.	**T**	.	.	.	.	.	.	.
KR559491	Colombia	.	.	**A**	.	.	.	.	.	.	.	.	.	.	.	**T**	.	.	.	.	.	.	.
KX496989	Colombia	.	.	**A**	.	T	.	.	.	.	.	.	.	.	.	.	.	.	.	.	.	.	.

Shaded polymorphisms: non-synonymous substitutions.

Supplementary Table S4. Set of primers for genome amplification (Stapleford *et al*. 2016)

	Primer	Sequence (5’--3’)	Genome Region	Size (pb)
1	1F	CACGTAGCCTACCAGTTTCTTA	17- 2113	2096
	nsP2-2112J-R	TTCTTGCAGCATCTTCTCTGGTCCA	5’ UTR – nsP2	
2	1F	CACGTAGCCTACCAGTTTCTTA	17- 872	855
	2R	ATGGAACACCGATGGTAGGTG	5’ UTR–nsP1	
3	3F	AACCCCGTTCTAGTACAACGC	617-2679	2081
	4R	CGGCATGTTGTACTCATTCG	nsP1–nsP2	
4	5F	CGAATTCGACAGCTTTGTAG	1411-3590	2179
	6R	GCACATGATGTCCGTTTATC	nsP1–nsP2	
5	7F	GACCAAGACTGAAAGTTGTAC	2490-4735	2237
	8R	CCACATAGTATGTATCTCTGC	nsP2–nsP3	
6	9F	GCGTACTGGGACGTAAGTTTA	3897-5824	1926
	10R	GGACGCACTCTCCTGGAGTTTC	nsP2–nsP3	
7	11F	CTGTACGGGAAGTGAGTATGAC	5064-6949	1903
	12R	CATACCGGATTTCATCATAGC	nsP3–nsP4	
8	13F	GGAGACGCCGTTTTAGAAACG	6748-8021	1283
	14R	CGCTTGAAGGCCAATTTGGCC	nsP4–capside	
9	15F	GCAGAGAGAGAATGTGCATG	7856-9799	1945
	16R	CCGCTTTAGCTGTTCTAATGC	capside- E2	
10	17F	GGAACTACCTTGCAGCACGTAC	8976-10989	2008
	18R	GGCGTTAGTCATCGAGTGCAC	E2–E1	
11	19F	GTACAGCAGAGTGTAAGGACCA	10172-11993	1821
	22R	AACATCTCCTACGTCCCTATGG	E1–3’UTR	
12	21F	GAACATGCCTATCTCCATCGAC	10803-11993	1196
	22R	AACATCTCCTACGTCCCTATGG	E1–3’UTR	

Supplementary Table S5. CHIKV whole genome sequences used in phylogenetic analyzes. The strain name, GenBank accession number, country, collection date and host of the sequences used in phylogenetic analyses.

Strain Name	GenBank Accession	Country (Imported)	year	GenBank Host
CHIKV-13-112A	AB860301	Philippines	2013	*Homo sapiens*
MY003IMR/06/BP	EU703760	Malaysia	2006	*Homo sapiens*
0706aTw	FJ807897	Indonesia (Taiwan)	2007	*Homo sapiens*
MY/06/37348	FN295483	Malaysia	2006	*Homo sapiens*
MY/06/37350	FN295484	Malaysia	2006	*Homo sapiens*
NC/2011-568	HE806461	New Caledonia	2011	*Homo sapiens*
PO731460	HM045788	India	1973	*Homo sapiens*
PhH15483	HM045790	Philippines	1985	*Homo sapiens*
JKT23574	HM045791	Indonesia	1983	*Homo sapiens*
CO392-95	HM045796	Thailand	1995	*Homo sapiens*
RSU1	HM045797	Indonesia	1985	*Homo sapiens*
3412-78	HM045808	Thailand	1978	*Homo sapiens*
TH35	HM045810	Thailand	1958	*Homo sapiens*
Gibbs 63-263	HM045813	India	1963	*Homo sapiens*
1455-75	HM045814	Thailand	1975	*Homo sapiens*
chik-sy	KF318729	China	2012	*Homo sapiens*
LEIV-CHIKV/Moscow/1/2013	KF872195	Indonesia (Russia)	2013	*Homo sapiens*
3807	KJ451622	Micronesia	2013	*Homo sapiens*
99659	KJ451624	British Virgin Islands	2014	*Homo sapiens*
Yap 13-2039	KJ689452	Micronesia	2013	*Ae. Aegypti*
DH130003	KM673291	Indonesia (Germany)	2012	*Homo sapiens*
M129	KM923920	Malaysia	2007	*Macaca fascicularis*
AMA2798/H804298	KP164567	Brazil	2014	*Homo sapiens*
PER10/H803609	KP164571	Dominican republic (Brazil)	2014	*Homo sapiens*
TR206/ H804187	KP164572.1	Guadalupe (Brazil)	2014	*Homo sapiens*
InDRE 51CHIK	KP851709	Mexico	2014	*Homo sapiens*
InDRE 4CHIK	KP851710	Caribe (Mexico)	2014	*Homo sapiens*
VE54_20	KR046231	Trinidad and Tobago	2014	*Homo sapiens*
VE55_4	KR046234	Trinidad and Tobago	2014	*Homo sapiens*
PR-S6	KR264951	Puerto Rico	2014	*Homo sapiens*
WHCHK3	KR559472	El Salvador	2014	-
WHCHK4	KR559473	French Polynesia	2015	-
WHCHK5	KR559474	Puerto Rico	2014	-
WHCHK10	KR559479.1	Dominican Republic	2014	-
WHCHK12	KR559481	Guatemala	2014	-
WHCHK15	KR559484	El Salvador	2014	-
WHCHK16	KR559485	Virgin Islands	2014	-
WHCHK17	KR559486	Panama	2014	-
WHCHK18	KR559487	Honduras	2014	-
WHCHK19	KR559488	Honduras	2014	-
WHCHK20	KR559489	Jamaica	2014	-
WHCHK21	KR559490	Guyana	2014	-
WHCHK22	KR559491	Colombia	2014	-
WHCHK23	KR559492	Saint Lucia	2014	-
WHCHK24	KR559493.1	American Samoa	2014	-
WHCHK25	KR559494	Virgin Islands	2014	-
WHCHK27	KR559496	Guyana	2014	-
WHCHK28	KR559497.1	St.Barts	2014	-
CPCC007800Y01	KT308159	Philippines	2012	*Homo sapiens*
CPCC017800Y01	KT308160	Philippines	2012	*Homo sapiens*
CPCC065200Y01	KT308161	Philippines	2012	*Homo sapiens*
CPCC083400Y01	KT308162	Philippines	2012	*Homo sapiens*
CPCC095700Y01	KT308163	Philippines	2012	*Homo sapiens*
CH0008	KT327163	Mexico	2014	*Homo sapiens*
241	KU365372.1	Colombia-Sincelejo	2014	*Homo sapiens*
246	KU365373.1	Colombia-Sincelejo	2014	*Homo sapiens*

257263	KU365374	Venezuela	2014	*Homo sapiens*
CHIKV/Homo sapiens/THA/6307-88/1988	KX262988	Thailand	1988	*Homo sapiens*
CHIKV/Homo sapiens/SXM/H-20235-STMARTIN-2013/2003	KX262991	Saint Martin	2013	*Homo sapiens*
CHIKV/Homo sapiens/GLP/YO-111213/2014	KX262992	Guadeloupe	2014	*Homo sapiens*
CHIKV/Homo sapiens/GUF/YO-123223/2014	KX262994	French Guiana	2014	*Homo sapiens*
Homo sapiens/COL/UF-1/2016	KX496989	Colombia-Bogotá (USA)	2016	*Homo sapiens*
N594	KY272962	Dominican Republic	2014	*Homo sapiens*
Haiti-5/2014	KY415980	Haiti	2014	*Homo sapiens*
Haiti-10/2014	KY415985	Haiti	2014	*Homo sapiens*
1.406.523	KY435455.1	Anguilla	2014	*Homo sapiens*
14.06350	KY435456	Suriname	2014	*Homo sapiens*
1.406.252	KY435457	Montserrat	2014	*Homo sapiens*
1.405.085	KY435459	Cayman Island	2014	*Homo sapiens*
1.405.081	KY435460	Cayman Island	2014	*Homo sapiens*
14.04425	KY435464	Barbados	2014	*Homo sapiens*
14.03562	KY435469	Grenada	2014	*Homo sapiens*
1.402.961	KY435470	Bahamas	2014	*Homo sapiens*
14.02585	KY435471	Turks and Caicos Islands	2014	*Homo sapiens*
1.402.217	KY435477	Guyana	2014	*Homo sapiens*
1.401.526	KY435479	Antigua y Barbuda	2014	*Homo sapiens*
14.01152	KY435482	Saint Kitts and Nevis	2014	*Homo sapiens*
1.400.686	KY435483.1	Anguilla	2014	*Homo sapiens*
14.00448	KY435484	Dominica	2014	*Homo sapiens*
Homo Sapiens /USA/CKVHHL_26/2014	KY680351.1	USA	2014	*Homo sapiens*
CHIKV/Homo sapiens/USA/CKVHL_22/2014	KY680369	USA	2014	*Homo sapiens*
CHIKV/Homo sapiens/USA/CKVHL_67/2014	KY680404	USA	2014	*Homo sapiens*
Homo Sapiens /NIC/4912.12.A.1 /2015	KY703930.1	Nicaragua	2015	*Homo sapiens*
CHIKV/Homo sapiens/NIC/1773.1C/2014	KY703969	Nicaragua	2014	*Homo sapiens*
CHIKV/Homo sapiens/NIC/13724-15/2015	KY703994	Nicaragua	2015	*Homo sapiens*
SGEHICH02971Y13	KY883764	Singapore	2013	*Homo sapiens*
Chikv/Hu/Colombia/NIID02/2015	LC259090.1	Colombia (Japon)	2015	*Homo sapiens*
M100	LN898093	Martinique	2013	*Homo sapiens*
G103	LN898099	Guadeloupe	2014	*Homo sapiens*
M105	LN898104	Martinique	2014	*Homo sapiens*
M111	LN898112	Martinique	2014	*Homo sapiens*
JFRO_07	MF001511	Puerto Rico	2015	*Homo sapiens*
Angola M2022	HM045823.1	Angola	1962	-
KPA15	HQ456254	Kenya-Mombasa	2004	*Homo sapiens*
LAMU33	HQ456255	Kenya-Lamu	2004	*Homo sapiens*
BHI3745/H804709	KP164570	Brazil	2014	*Homo sapiens*
Vereeniging'	HM045792	South Africa	1956	*Homo sapiens*
SAH2123	HM045795	South Africa	1976	*Homo sapiens*
AR 18211	HM045805	South Africa	1976	*Aedes furcifer*
Ross low-psg	HM045811	Tanzania	1953	*Homo sapiens*
A301	HM045821	Senegal	1963	*Chiroptera*
DakAr B 16878	HM045784	Central African Republic	1984	*Anopheles (Ceilia) funestus*
LSFS	HM045809	The Democratic Republic of the Congo	1960	*Homo sapiens*
UgAg4155	HM045812	Uganda	1982	*Homo sapiens*
HB78	HM045822	Central African Republic	1978	*Homo sapiens*
BR33	KX228391	Brazil - Pernambuco	2016	*Homo sapiens*
ArB6445	KY038946	Central African Republic-Bozo	1975	*Aedes opok*
HB84P07	KY038947	Central African Republic	1983	*Homo sapiens*

C302F/2016/BR	KY055011	Brazil -Arcaju	2016	*Aedes aegypti*
05-209	AM258991	Seychelles	2005	*Homo sapiens*
LR2006_OPY1	DQ443544.2	Reunion	2006	*Homo sapiens*
BNI-CHIKV_899	FJ959103	Mauritius	2006	*Homo sapiens*
IMTSSA6424C	FR717337	Reunion	2005	*Homo sapiens*
L2	KF283986	Comoros	2005	*Homo sapiens*
S7	KF283987.1	Comoros: Grande Island	2005	-
OPY6	KP003807	France	2006	*Homo sapiens*
MADOPY1	KP003808	Madagascar (Francia)	2006	*Homo sapiens*
OPY4	KP003809	Mayotte	2006	*Homo sapiens*
LR2006_OPY1	KT449801	Reunion	2006	*Homo sapiens*
DRDE-07	EU372006	India	2007	*Homo sapiens*
LKRGCH1507	FJ445428	Sri Lanka	2007	*Homo sapiens*
SGEHICHS277108	FJ445510	Singapore	2008	*Homo sapiens*
LK(PB)CH5308	FJ513635	Sri Lanka	2008	*Homo sapiens*
0611aTw	FJ807896	Singapore (Taiwan)	2006	*Homo sapiens*
0810aTw	FJ807898	Bangladesh (Taiwan)	2008	*Homo sapiens*
MY/08/068	FN295487	Malaysia	2008	*Homo sapiens*
SL15649	GU189061	Sri Lanka	2006	*Homo sapiens*
SD08Pan	GU199351	Sri Lanka (China)	2008	*Homo sapiens*
CU-Chik10	GU301780	Thailand	2008	*Homo sapiens*
CU-Chik683	GU301781	Thailand	2009	*Homo sapiens*
CU-Chik_OBF	GU908223	Thailand	2009	*Aedes albopictus*
GD115	HQ846356	China	2010	*Homo sapiens*
IND-06-Guj	JF274082	India	2006	*Homo sapiens*
V1024313_KH11_PVH	JQ861257	Cambodia	2011	*Homo sapiens*
Yem-11	KC614648	Yemen	2011	*Aedes aegypti (mosquito)*
NL10/152	KC862329	Indonesia	2010	*Homo sapiens*
Myanmar/D136/2009	KF151175	Myanmar	2009	*Homo sapiens*
10Mdy105	KF590567	Myanmar	2010	*Homo sapiens*
BK57	KJ579185	Thailand	2013	*Homo sapiens*
CHIKV_STMWG02	KJ679578	India	2011	*Homo sapiens*
RGCB730/09	KJ796844	India	2009	*Homo sapiens*
StBI	KP003811	Italy	2007	*Homo sapiens*
RGCB855/10	KT336778	India	2010	*Homo sapiens*
RGCB1259/12	KT336779	India	2012	*Homo sapiens*
RGCB1431/13	KT336782	India	2013	*Homo sapiens*
CHIKV/Homo sapiens/ITA/Bianchi/2007	KX262989	Italy	2007	*Homo sapiens*
JHCK96	KX619424	India	2015	*Homo sapiens*
119067	KY057363	India	2016	*Homo sapiens*
IN16C1	KY751908	India (Australia)	2016	*Homo sapiens*
hk02	MF499120	India (Hong Kong)	2016	*Homo sapiens*
Pakistan-04/2016	MF774614	Pakistan	2016	*Homo sapiens*
Pakistan-07/2016	MF774617	Pakistan	2016	*Homo sapiens*
37997	AY726732	Senegal	1983	*Aedes furcifer*
PM2951	HM045785	Senegal	1966	*Aedes aegypti*
IbH35 (TVP1337)	HM045786	Nigeria	1964	*Homo sapiens*
SH2830	HM045798	Senegal	1966	*Homo sapiens*
IbAn4824	HM045807	Nigeria	1965	*sentinel mouse*
ArD 30237	HM045815	Senegal	1979	*Ae. luteocephalus*
HD 180760	HM045817	Senegal	2005	*Homo sapiens*
ArA 2657	HM045818	Cote dIvoire	1981	*Ae. luteocephalus*
ArD 93229	HM045819	Senegal	1993	*Ae. dalzieli*
ArA 30548	HM045820	Cote dIvoire	1993	*Aedes africanus*
CHIKV/Aedes furcifer/SEN/37950/1983	KX262995	Senegal	1983	*Ae. furcifer*
